# Hydrodynamic constraints on the energy efficiency of droplet electricity generators

**DOI:** 10.1038/s41378-021-00269-8

**Published:** 2021-06-21

**Authors:** Antoine Riaud, Cui Wang, Jia Zhou, Wanghuai Xu, Zuankai Wang

**Affiliations:** 1 State Key Laboratory of ASIC and System, School of Microelectronics, Fudan University, Shanghai, 200433 China; 2 Department of Mechanical Engineering, City University of Hong Kong, Hong Kong, 999077 China

**Keywords:** Electrical and electronic engineering, Environmental, health and safety issues, Physics

## Abstract

Electric energy generation from falling droplets has seen a hundred-fold rise in efficiency over the past few years. However, even these newest devices can only extract a small portion of the droplet energy. In this paper, we theoretically investigate the contributions of hydrodynamic and electric losses in limiting the efficiency of droplet electricity generators (DEG). We restrict our analysis to cases where the droplet contacts the electrode at maximum spread, which was observed to maximize the DEG efficiency. Herein, the electro-mechanical energy conversion occurs during the recoil that immediately follows droplet impact. We then identify three limits on existing droplet electric generators: (i) the impingement velocity is limited in order to maintain the droplet integrity; (ii) much of droplet mechanical energy is squandered in overcoming viscous shear force with the substrate; (iii) insufficient electrical charge of the substrate. Of all these effects, we found that up to 83% of the total energy available was lost by viscous dissipation during spreading. Minimizing this loss by using cascaded DEG devices to reduce the droplet kinetic energy may increase future devices efficiency beyond 10%.

## Introduction

Droplet electricity generators (DEG) are designed to harvest the kinetic energy of rain droplets to power small wireless sensors. Despite a 100-fold increase in efficiency over the past few years^1–3^, even state-of-the-art devices only recover 10% of the kinetic energy of water^4^, as opposed to the nearly 100% efficiency achieved by hydroelectric dams.

**Fig. 1:**
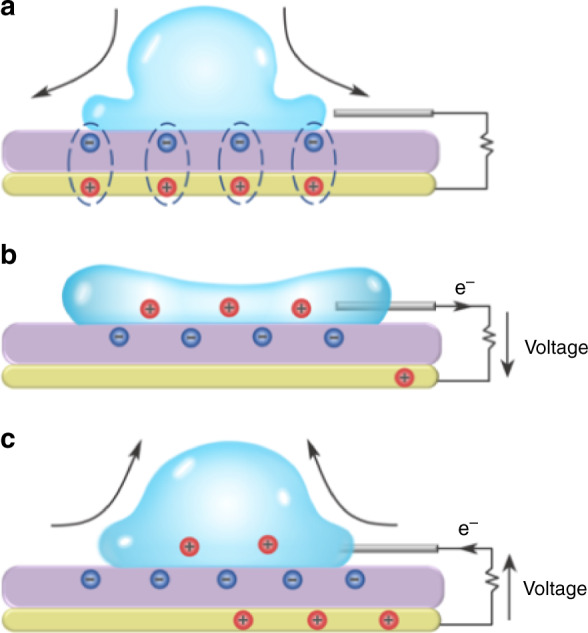
Droplet electric generator with charge circulation^1,4,13^. **a** The substrate is initially charged by the impingement of many droplets or other electrical forcing^13^ and forms charge pairs on each side of the substrate. **b** Upon contact, the substrate capacitor is discharged through the load and the liquid. **c** During the recoil, trapped charges in the polymer are left behind, so that positive charges move back to the ITO to restore the charge pairs

Unlike dams, which extract energy from the mechanical work of water on the hydro-turbines, DEG, and more broadly triboelectric nanogenerators (TENG) harvest energy from charges accumulated on surfaces which are then used to drive an electric current through an external circuit by electrostatic induction^5^. In the case of DEG, the charges are spontaneously created by water at the surface of polymers^6–10^ by an electron-mediated contact electrification^11,12^. The process can be intensified by applying a voltage across the polymer layer^13^. In the latest studies, a grounded metallic electrode is placed underneath the polymer and is connected to a small metallic strip on the top (see Fig. 1). According to the present understanding^2,14^, this sandwiched structure then behaves as a biased capacitor. Upon contact with water, the capacitor is discharged through the load, which releases the electrostatic energy that was stored previously^2^. Meanwhile, mobile charges accumulate at the water-polymer interface. When the droplet recedes, those charges are detached from the interface and forced to return to the bottom electrode^4^. While this model predicts the transfer of charges through the DEG with a remarkable accuracy^2^, it does not consider the hydrodynamic side of the picture. Yet, the harvested electrical energy accounts at best for 10% of the initial droplet energy, meaning that, in our present understanding, at least 90% of the droplet energy is unaccounted for.

A comprehensive DEG model would consider (i) the electrical process at play, (ii) the hydrodynamics (iii) the electrochemical charge stability and (iv) the electrohydrodynamic coupling. Since only 10% of the DEG energy is electrical^4^, we neglect the electrohydrodynamic coupling and focus mainly on the hydrodynamic process. Indeed, droplet impacts have been shown to obey the same dynamics regardless of the susbstrate charge^1^. In this simplified view, the DEG hydrodynamics are exactly those of a droplet impacting an inclined plane. Even in this elementary picture, three effects compete to dominate the droplet dynamics: inertia, surface forces, and viscous dissipation. For a spherical droplet of radius *a*, density *ρ*, viscosity *μ*, surface tension *γ* falling at a velocity *U*_0_, the ratio of kinetic energy to viscous work is approximately the Reynolds number ${\rm{Re}}=\frac{\rho a{U}_{0}}{\mu }$, while the ratio of kinetic energy to surface energy is connected to the Weber number ${\rm{We}}=\frac{\rho {{U}_{0}}^{2}a}{\gamma }$. During impacts, the liquid spreads into a thin lamella on the solid where most of the energy conversion occurs^15^. It was noticed quite early that a large fraction of the droplet energy is lost during impact^16^. This loss was attributed to viscous dissipation within the lamella^16–18^ until experiments of low-viscosity droplet impacts on super-hydrophobic and slippery substrates suggested that a large fraction of the energy was actually converted to internal kinetic energy (akin to turbulence)^19–21^. Several recent works have since attempted to bridge the gap between these two regimes^15,22,23^. The numerical simulations of Wildeman et al.^15^ are of particular interest for this study, as they show the location of viscous losses within the droplets during impacts, and quantify the fraction of energy dissipated as viscous work and internal kinetic energy over the entire spreading step with no-slip and free-slip boundary conditions. According to this model, nearly half of the initial kinetic energy of low-viscosity droplets is lost as viscous dissipation in the lamella during spreading, regardless of the slip length, impact velocity and fluid viscosity.

The stability of the lamella formed during the impact is also a key concern for DEG. Intuitively, a splashing droplet releases some surface and kinetic energy in the form of ejected daughter droplets. Lamella breakup depends on a competition between a destabilizing suction and lubrication forces that lift it from the substrate, and a restoring capillary force that pulls the liquid back into the bulk of the droplet^24^. When the restoring force is overwhelmed by the other two, the lamella detaches and breaks into smaller droplets, resulting in splashing^25^. Even if the lamella remains stable and recedes, the liquid film itself may have thinned past its own stability limit and rupture into lower-energy liquid islands^26^.

In this paper, we investigate the consequences of the hydrodynamic phenomena on the operating conditions and efficiency of DEG devices. Even though most DEG devices can provide energy during the spreading of the drop^1,2,4^, it was experimentally observed that the DEG energy output peaks when the electrode is placed far enough from the droplet impact such that the electrical contact is established at maximum droplet spread^1,2^. Therefore, we will restrict our analysis to these situations where the droplet spreads to its maximum extent before touching the electrode. In the absence of electrical contact, the liquid provides no electrical energy during the spreading phase, and instead the totality of the energy is obtained during the receding of the liquid film. This is supported by a thought experiment where one would drop a neutral conducting disc on the DEG. The biased capacitor would release the same amount of energy without any work from the disc. However, one would need to overcome the Coulomb force between the mobile charges induced in the disc and the underlying polymer to detach the disc from the DEG, thereby attesting of an electromechanical conversion during recoil. We note that this energy is first stored as electrostatic energy in the polymer until a new disc is placed to release it, thereby completing the energy production cycle. The paper is organized as follows. The first part of the paper discusses the hydrodynamic limits of droplet impacts and illustrates these conditions with simple expressions from the extensive literature on normal droplet impacts^15–21,23–29^. Starting from the total energy available from a falling droplet, we will first estimate the maximum impact velocity allowed for DEG devices depending on the droplet volume, then evaluate how this energy is converted into surface energy and turbulent/viscous losses during the spreading phase, followed by the recoil phase. In the second part of the paper, we complement this hydrodynamic picture with the electrical model of Wu et al.^2^ to quantify the energy efficiency of DEG devices. We use experimental data from previous studies^1,2,4^ to explore various operating conditions in a realistic setting (oblique impacts) and point out to possible improvements for this technology.

## Results and discussion

### Fluid mechanics model

#### Available energy

A falling droplet combines a kinetic energy ${{\mathcal{K}}}_{0}=\frac{2\pi }{3}{a}^{3}\rho {{U}_{0}}^{2}$ and surface energy ${{\mathcal{V}}}_{0}=4\pi {a}^{2}\gamma$. Neglecting gravity, the minimum energy of a liquid in contact with a solid surface is obtained when the spherical droplet intersects with the solid surface at the Young contact angle $\cos \theta =\frac{{\gamma }_{sv}-{\gamma }_{sl}}{\gamma }$. According to volume conservation, this yields^30^:

1 $${{\mathcal{V}}}_{\text{eq}}=\gamma {A}_{\text{eq,cap}}-\gamma \cos \theta {A}_{\text{eq,base}}$$

2 $$\,\text{with:}\,\ \ {A}_{\text{eq,cap}}=2\pi {{R}_{\text{eq}}}^{2}(1-\cos \theta )$$

3 $${A}_{\text{eq,base}}=\pi {{R}_{\text{eq}}}^{2}\,{\sin }^{2}\theta$$

4 $$\,\text{and}\,\ \ {R}_{\text{eq}}=\frac{{2}^{2/3}a}{{(2-3\cos \theta +{\cos }^{3}\theta )}^{1/3}}$$

where *A*_eq,cap_ and *A*_eq,base_ are the cap and base surface area of the droplet at equilibrium, and *R*_eq_ is its radius of curvature. This ideal situation is never achieved in practical DEG devices. Therefore, the maximum energy available is obtained by subtracting this lowest possible energy from the initial energy:

5 $${{\mathcal{E}}}_{\max }={{\mathcal{K}}}_{0}+{{\mathcal{V}}}_{0}-{{\mathcal{V}}}_{\text{eq}}$$

#### Maximum impact velocity

The impact velocity is limited by two factors. On the one hand, the droplet may splash, but even without splashing, the film formed by the impacted droplet may still become unstable and rupture. We first recall the splashing conditions according to Riboux and Gordillo^25^. While their study focuses on normal impacts, they mainly discuss the local dynamics of the lamella formed during the droplet impact, which suggests that, at least qualitatively, the splashing mechanism evidenced by these authors may also be relevant for oblique impacts.

When a droplet impacts a solid surface, it forms a lamella that may recoil or break into droplets if splashing occurs. To splash, the lamella must satisfy two conditions: (i) the liquid sheet must be ejected from the solid and (ii) the growing rim at the edge of the lamella must not reconnect with the solid afterwards. Interpreting condition (i) as a force balance yields the ejection ejection time ${T}_{e}={t}_{e}\frac{a}{{U}_{0}}$^25^ from the real positive root $\sqrt{{t}_{e}}$ of the fourth-order polynomial:

6 $$\frac{\sqrt{3}}{2{\rm{Re}}}+\frac{\sqrt{{t}_{e}}}{We}=1.1{{t}_{e}}^{2}$$

Upon ejection, the lamella lifts off with a vertical velocity *V*_*v*_. However, the ejected film also recoils with a speed *V*_*r*_ due to surface tension, which forms a fast-growing rim that may eventually reconnect with the solid, thereby preventing splashing. A mass balance shows that the rim grows at a rate ${b}_{\max }{V}_{r}$, which yields the second splashing condition:

7 $${V}_{v}\ge {b}_{\max }{V}_{r}$$

where the constant ${b}_{\max }=0.14$ was determined experimentally by Riboux and Gordillo^25^. Lamella recoil speed *V*_*r*_ and vertical speed *V*_*v*_ at the ejection time are given by:

8 $${V}_{r}=\sqrt{2\gamma /\rho {H}_{t}}$$

9 $${V}_{v}=\sqrt{\frac{\ell }{\rho {H}_{t}}}$$

10 $${\text{with}}\,\ \ \ell ={K}_{l}{\mu }_{g}{V}_{t}+{K}_{u}{\rho }_{g}{{V}_{t}}^{2}{H}_{t}$$

11 $${\text{and}}\,\ \ {H}_{t}=(a\sqrt{12}/\pi ){{t}_{e}}^{3/2}$$

Here, *H*_*t*_ is the lamella film height at *T*_*e*_, and ${K}_{l}\simeq -\left[6/{\tan }^{2}\alpha \right]({\mathrm{log}}\,(19.2{\lambda }_{g}/{H}_{t})-{\mathrm{log}}\,(1+19.2{\lambda }_{g}/{H}_{t}))$ and *K*_*u*_ = 0.3 are two hydrodynamic coefficients for the suction and lubrication forces that make up the lift force *ℓ*. *ρ*_*g*_, *μ*_*g*_ and *λ*_*g*_ are the gas phase density, dynamic viscosity and mean-free path.

Even in the absence of splashing, the liquid film formed after impact may still rupture. Surprisingly, even though instability of static liquid films is a well-studied topic^31–33^, film rupture immediately after droplet impact has received much less attention than the more spectacular splashing. Diman and Chandra^26^ have studied the disintegration of liquid films formed after high-speed normal collision between a droplet and a wall. While their analysis is rather involved, and depends on the liquid-solid contact angle and the size of defects that trigger the liquid film instability, experimental evidence over a range of contact angles, surface roughness and liquids suggest that the liquid film will rupture if the droplet impacts a solid wall above some critical Reynolds number ${{\rm{Re}}}_{c}\simeq 5000$:

12 $${\rm{Re}}\ge {{\rm{Re}}}_{c}$$

**Fig. 2:**
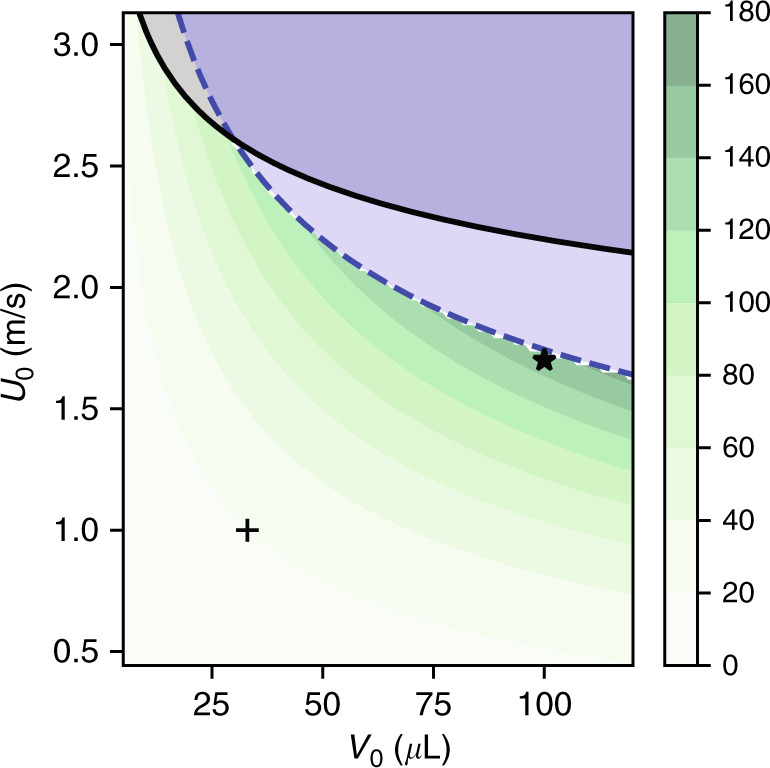
The available energy and the limiting velocities for splashing and film rupturing. Maximum energy ${{\mathcal{E}}}_{\max }$ (in μJ) available from droplets impacting perpendicular surfaces as given by Eq. (5). The splashing (solid line) and film-rupturing (dashed line) limits are given by Eqs. (7) and (12). The markers + and ⋆ represent the experiments of Wu et al.^2^ (energy available 19 μJ) and Xu et al.^1^ (energy available 151 μJ), respectively

The available energy and the limiting velocities for splashing (Eq. (7)) and film rupturing (Eq. (12)) are shown in Fig. 2. For the sake of simplicity, this figure is computed for deionized (DI) water but would be almost identical for tap water, sea water, rain water and 100 mM NaCl water solutions as they share very similar mechanical properties, as discussed in Supplementary Information. Note that smaller droplets tend to splash first while larger ones may not splash but their liquid film will rupture nonetheless. The largest theoretical amount of energy while maintaining droplet integrity is obtained for the largest droplets at velocities close to 1.7 m/s, remarkably close to Xu et al.^1^.

#### Efficiency during spreading and receding

Depending on the impact speed, a sizable fraction of the available energy (Eq. (5)) may be dissipated into turbulent kinetic energy and viscous work during the droplet spreading. This yields the conversion efficiency *η*_*s*_:

13 $${\eta }_{s}=\frac{{{\mathcal{V}}}_{\max }}{{{\mathcal{E}}}_{\max }}$$

with ${{\mathcal{V}}}_{\max }$ the resulting surface energy at maximum spread ${{\mathcal{V}}}_{\max }=\gamma (1-\cos \theta ){A}_{\max }$, where the surface energy ${A}_{\max }$ may be determined from experimental or simulated droplet impacts, or using simplified models (see refs. ^18^^,^^23^ for critical reviews on normal impacts, and ref. ^22^ for oblique impacts). Among these models, Pasandideh-Fard et al.^17^ have provided a simple and accurate estimate of the maximum spreading diameter of droplets impinging on a perpendicular surface:

14 $${a}_{\max }=a\sqrt{\frac{{\rm{We}}+6}{\frac{3}{2}(1-\cos \theta )+4{\rm{We}}\sqrt{\frac{2}{{\rm{Re}}}}}}$$

**Fig. 3:**
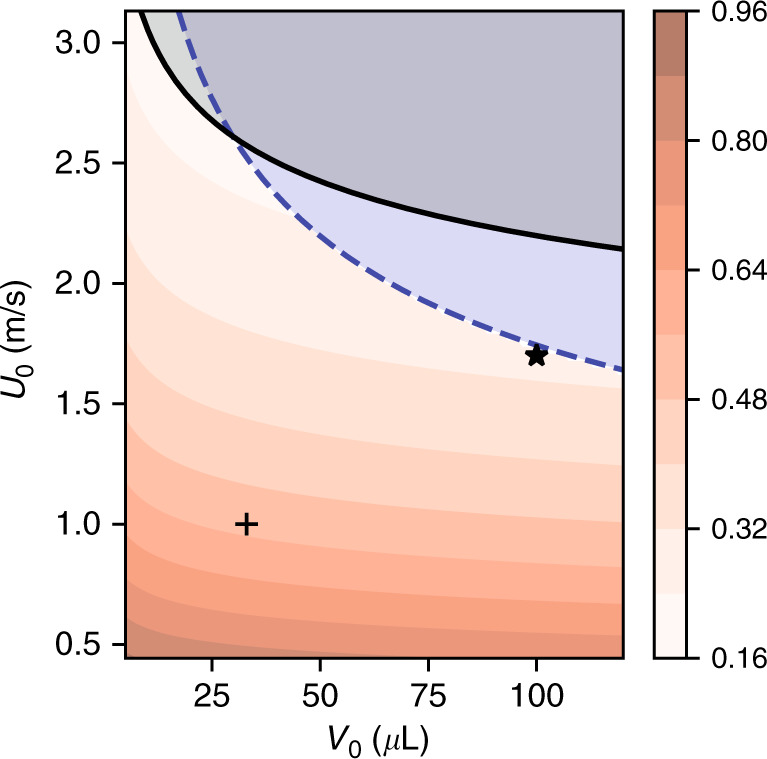
Mechanical to kinetic conversion efficiency *η*_*s*_ (%) according to Eq. (14) for droplets impacting perpendicular surfaces. The splashing (solid line) and film-rupturing (dashed line) limits are given by Eqs. (7) and (12). The markers + and ⋆ represent the experiments of Wu et al.^2^ (spreading efficiency 17%) and Xu et al.^1^ (spreading efficiency 30%), respectively

In spite of being derived for highly viscous fluids only, this formula was empirically found to work well for low-viscosity fluids as well^18,23^. We refer the reader to Wildeman et al.^15^ for a more physically-sound model at low viscosity. The resulting energy efficiency obtained by combining Eqs. (13) and (14) is shown in Fig. 3. Similarly to Fig. 2, this figure is computed for DI water but would look essentially the same for other diluted water solutions.

During the recoil phase, the surface energy ${{\mathcal{V}}}_{\max }$ is split into 3 terms: the viscous work during recoil ${{\mathcal{W}}}_{r}$, the electrical work during recoil ${{\mathcal{W}}}_{e}$ and the final mechanical energy remaining in the droplet ${{\mathcal{E}}}_{\infty }$:

15 $${{\mathcal{V}}}_{\max }={{\mathcal{W}}}_{r}+{{\mathcal{W}}}_{e}+{{\mathcal{E}}}_{\infty }$$

Experiments with water drops bouncing on super-hydrophobic substrates^21,29^ suggest that the viscous dissipation is small during the recoil step, even at high impact velocity, and that most of the surface energy is either restored as external kinetic energy or vibration energy. This is confirmed by the following rough estimate of the droplet viscous dissipation. We first assume that the recoil speed scales as ${U}_{r}={a}_{\max }/{\tau }_{r}$, with *τ*_*r*_ the contact time between the droplet and the substrate. For super-hydrophobic substrates, the contact time, that is a negligible spreading time plus the receding time, is insensitive to the impact velocity^19,27^. Hence, the recoil time scales proportionally to ${\tau }_{r}=\pi {\tau }_{h}/\sqrt{2}$ with ${\tau }_{h}=\sqrt{\frac{\rho {a}^{3}}{\gamma }}$ the oscillation period of a levitated drop^27,34^. Using *U*_*r*_ as the characteristic recoil velocity, we then compute the volumetric shear rate similarly to Pasandideh-Fard^17^ and obtain a coarse estimate of the viscous work during recoil:

16 $${{\mathcal{W}}}_{r}\simeq {\tau }_{r}\mu {\left(\frac{{U}_{r}}{\delta }\right)}^{2}{V}_{0}$$

17 $$\,\text{with:}\,\ \ \delta =\min ({h}_{\text{bl}},{h}_{\text{film}})$$

18 $${h}_{\text{bl}}=\sqrt{\frac{2\mu {\tau }_{r}}{\rho }}$$

19 $${h}_{\text{film}}=\frac{{V}_{0}}{{A}_{\max }}$$

where *h*_bl_ and *h*_film_ are the hydrodynamic boundary layer and film thickness during recoil.

### Electrical model

#### Biased capacitor model

By analogy with a biased capacitor, Wu et al.^2^ have derived the following equation for the charge *q* driven through the load by the droplet motion:

20 $$\frac{dq}{dt}=\frac{1}{R{c}_{p}}\left(\sigma -\frac{q}{A}\right)$$

with *σ* the surface charge of the polymer, *c*_*p*_ the areal capacitance of the polymer and *R* = *R*_*L*_ + *R*_*D*_ the total resistance of the circuit, including the droplet resistance *R*_*D*_ and the load *R*_*L*_. In the biased capacitor model, *A*(*t*) stands for the evolving area of the droplet, but the overlap area of charged polymer in contact with the droplet should be used instead when the polymer charge is non-uniform^4^. We note that this model considers the polymer charge at the time of the droplet impact, regardless of how this charge was generated (such as successive droplet impacts^1,35^ or external charging^4,13^).

The energy output reads ${{\mathcal{W}}}_{L}=\mathop{\int}\nolimits_{0}^{{t}_{c}}{R}_{L}{\left(\frac{dq}{dt}\right)}^{2}dt$, where the origin of time *t* = 0 is chosen at droplet contact, and *t*_*c*_ is the time when the droplet detaches from the electrode. Using integration by part and substituting Eq. (20) in this integral yields:

21 $${{\mathcal{W}}}_{L}={\eta }_{L}\left({{\mathcal{E}}}_{\text{stat}}-{{\mathcal{E}}}_{\text{dyn}}\right)$$

22 $$\,\text{with:}\,\ \ {\eta }_{L}=\frac{{R}_{L}}{R}$$

23 $${{\mathcal{E}}}_{\text{stat}}=\frac{\sigma q({t}_{c})}{{c}_{p}}$$

24 $${{\mathcal{E}}}_{\text{dyn}}=\frac{1}{2{c}_{p}}\mathop{\int}\nolimits_{0}^{{t}_{c}}\frac{\frac{d{q}^{2}}{dt}}{A}dt$$

Note that the right-hand side of Eq. (24) cannot be simplified immediately because there is no one-to-one correspondence between *A*(*t*) and *q*^2^(*t*). Eq. (21) is made of three parts: an efficiency factor *η*_*L*_ that accounts for the resistive losses in the liquid, an electrostatic energy ${{\mathcal{E}}}_{\text{stat}}$ and a contribution that depends on the dynamics of the charges and droplet geometry ${{\mathcal{E}}}_{\text{dyn}}$. Analytical integration of Eq. (20) (see Supplementary Information) shows that ${{\mathcal{E}}}_{\text{dyn}}$ is positive only if *q*^2^ decreases, meaning that this term acts as a generator only when charges are moving out of the droplet.

#### Available energy

The droplet exchanges electrical energy with the load twice. First during the discharge of the bottom electrode in the liquid, and then during the recoil step. As stated in the introduction, we restrict our analysis to cases where the droplet spreads to its maximum diameter before contacting the electrode, so that the discharge process goes without energy exchange between the droplet and the DEG: the liquid merely acts as a conductor to release the stored electrostatic energy. Although the discharge step requires no work from the droplet, the droplet-polymer interface becomes increasingly charged thereafter. During the recoil step, the liquid interface area shrinks such that the liquid-polymer interfacial capacitance decreases, which prompts charges to flow back to the bottom electrode. During charge separation, the dry polymer recovers its static charge, which builds up electrostatic energy that will be released by the next droplet. In order to reduce the contact area between oppositely charged surfaces, some electrowetting work must be provided to overcome the electrostatic energy^36,37^. When charges flow back, they provide resistive electrical work through the load but also through the liquid, thereafter called resistive losses:

25 $${{\mathcal{W}}}_{\text{R}}=(1-{\eta }_{L})\left({{\mathcal{E}}}_{\text{stat}}-{{\mathcal{E}}}_{\text{dyn}}\right)$$

Upon sufficient recoil, the droplet eventually detaches from the top electrode and the current stops flowing through the load. Integrating Eq. (20) shows that the static loss cannot be entirely eliminated unless the droplet area vanishes at *t*_*c*_ (see materials and methods). This can be achieved by using non-uniform surface charges^4^. While there is no constraint on the remaining amount of charges (missing charges can be provided from the electrical ground), a highly charged droplet will need to expend more energy to leave the substrate than a neutral one. Therefore, the less charge remains in the droplet, the more energy is available for the load. We will refer to the additional electrostatic expense as the static loss ${{\mathcal{W}}}_{\text{stat}}=\frac{{q}^{2}({t}_{\infty })}{2{c}_{p}A({t}_{\infty })},$ where *t*_*∞*_ indicates the time the droplet breaks away from the DEG substrate. By conservation of charge, *q*(*t*_*c*_) = *q*(*t*_*∞*_), and by conservation of energy the work provided to change the droplet surface area must compensate exactly the variation of ${{\mathcal{W}}}_{\text{stat}}$, therefore:

26 $${{\mathcal{W}}}_{\text{stat}}=\frac{{q}^{2}({t}_{c})}{2{c}_{p}A({t}_{c})}$$

### Main equations

In summary, the energy extracted from the DEG (${{\mathcal{W}}}_{L}$) is the maximum available energy ${{\mathcal{E}}}_{\max }$ minus the mechanical (${{\mathcal{W}}}_{\text{mech}}$) and electrical ${{\mathcal{W}}}_{\text{electr}}$ losses.

$${{\mathcal{W}}}_{L}={{\mathcal{E}}}_{\max }-{{\mathcal{W}}}_{\text{mech}}-{{\mathcal{W}}}_{\text{electr}}$$

The mechanical energy is dissipated during spreading, recoil, and droplet detachment:

$$\begin{array}{lll}\qquad\qquad\qquad{\text{spreading}}\, {\text{work}}\,{:}\quad&1-{\eta }_{s}&=1-\frac{{{\mathcal{V}}}_{\max }}{{{\mathcal{E}}}_{\max }}\\ \qquad\qquad\qquad\qquad{\text{recoil}}\, {\text{work}}\,{:}\quad&{{\mathcal{W}}}_{r}&\simeq {\tau }_{r}\mu {\left(\frac{{U}_{r}}{\delta }\right)}^{2}{V}_{0}\\ \;{\text{untapped}}\, {\text{mechanical}}\, {\text{energy}}\,{:}\quad&{{\mathcal{E}}}_{\infty }&\end{array}$$

Here, ${{\mathcal{V}}}_{\max }$ is obtained experimentally or empirically from the maximum droplet spread ${A}_{\max }$ and ${{\mathcal{W}}}_{r}$ is computed from Eq. (16). The untapped mechanical energy ${{\mathcal{E}}}_{\infty }$, attributed to the mechanical energy remaining in the escaping droplet, is obtained from Eq. (15) after computing the electrical work ${{\mathcal{W}}}_{e}={W}_{\text{electr}}+{{\mathcal{W}}}_{L}$. The electric losses are the resistive losses in the liquid and the electromechanical work needed to separate a charged droplet from a charged surface:

$$\begin{array}{lll}{\text{resistive}}\, {\text{losses}}\,{:}\,&{{\mathcal{W}}}_{\text{R}}&={\displaystyle\int\nolimits_{0}^{{t}_{c}}}{R}_{D}{\left(\frac{dq}{dt}\right)}^{2}dt\\ \,{\text{static}}\, {\text{charge}}\, {\text{losses}}\,{:}\,&{{\mathcal{W}}}_{\text{stat}}&=\frac{{q}^{2}({t}_{c})}{2{c}_{p}A({t}_{c})}\end{array}$$

The extracted energy reads:

$${{\mathcal{W}}}_{L}=\mathop{\int}\nolimits_{0}^{{t}_{c}}{R}_{L}{\left(\frac{dq}{dt}\right)}^{2}dt$$

where the charge *q*(*t*) traveling through the load is obtained experimentally or by numerically solving Eq. (20). A table of symbols is available in the Supplementary Information.

### Discussion

**Fig. 4:**
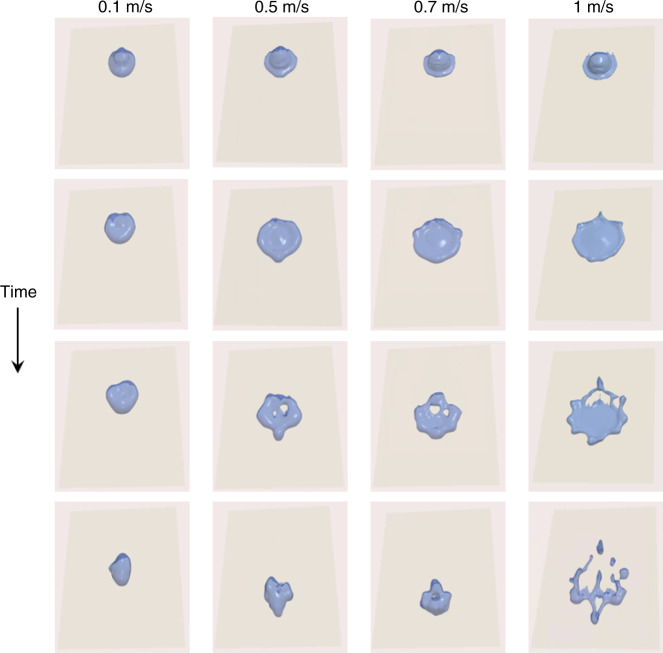
Simulations of droplet impact on Teflon. The liquid film becomes unstable for impact velocities above 1 m/s. See the materials and methods for the simulation parameters. The pictures timestamps are available in the Supplementary Information

Figure 2 suggests that large droplets yield the maximum energy within hydrodynamic stability limits. In order to get a more faithful picture under these optimized conditions, we have simulated in OpenFOAM the impact of 100 μl droplets on Teflon at various impact velocities (Fig. 4) with an impact angle of 45° (similar to the one used by Xu et al.^1^). We note that this angle has a limited influence on the performance of DEG devices^2^. In agreement with the stability bounds, we observe the onset of film rupture (and not splashing) when the impact velocity exceeds 1 m/s (${\rm{Re}}\simeq 2000$).

**Fig. 5:**
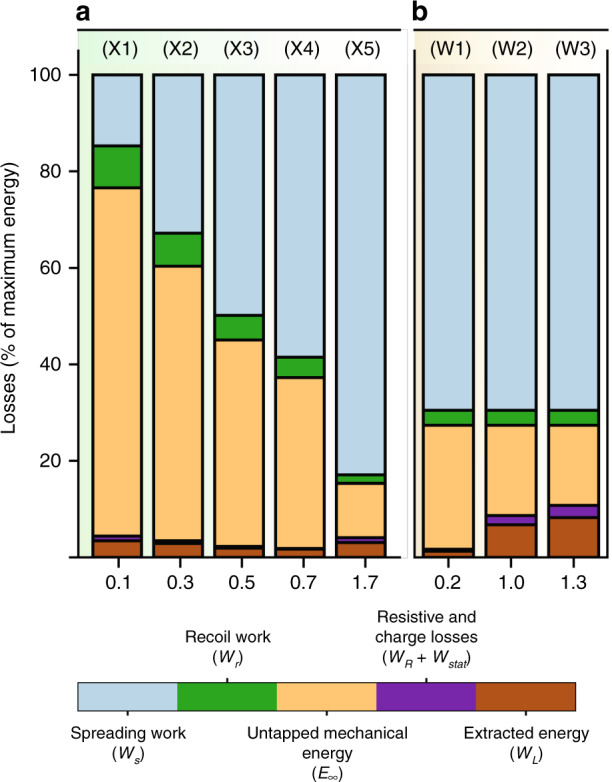
Energy losses at successive stages of the electricity generation as a percentage of the maximum energy ${{\mathcal{E}}}_{\max }$. **a** for 100 μL droplets with impinging speed ranging from 0.1 to 1.7 m/s, **b** for 33 μL droplets with varying initial electrical energy ${E}_{0}={A}_{\max }{\sigma }^{2}/(2{c}_{p})$. The symbols (X1..5) and (W1..3) indicate the experimental parameters according to Table 1

Based on DEG impact videos^1,2^ and our simulations, we evaluate the surface energy ${{\mathcal{V}}}_{\max }=\gamma (1-\cos \theta ){A}_{\max }$ available as the impinged droplet spreads to its maximum diameter. The viscous work during recoil is then deduced from Eq.(16). Similarly to previous studies^1,2^, we optimize the value of *R*_*L*_ to maximize the energy output at given *A*(*t*) (obtained from experimental videos or simulations). With these optimized parameters, we compute the energy generated per droplet, together with the static and resistive losses (Eqs. (21), (26) and (25)), which yields the total electrical energy available ${{\mathcal{W}}}_{e}$. The simulation parameters are given in Table 1 together with the final conversion efficiency, and the relative shares of each energy contribution are shown in Fig. 5.

**Table 1:** Parameters used for droplet electric generation loss analysis in Fig. 5

Case	Volume	Impact speed	Dielectric	Surface charge	A(t)	*E*_0_	${{\mathcal{E}}}_{\max }$	Extracted energy (model)	Extracted energy (experiment)
	(μl)	(m/s)		(mC/m^2^)		(μJ)	(μJ)	(%)	(%)
(X1)	100	0.1	PTFE (16 μm)	0.17	Simulated	0.2	7.3	3.43	/
(X2)	100	0.3	PTFE (16 μm)	0.17	Simulated	0.2	11.3	2.88	/
(X3)	100	0.5	PTFE (16 μm)	0.17	Simulated	0.3	19.2	1.91	/
(X4)	100	0.7	PTFE (16 μm)	0.17	Simulated	0.4	31.2	1.71	/
(X5)	100	1.7	PTFE (16 μm)	0.17	Experimental^1^	3.4	150.1	3.08	2.2
(W1)	33	1.0	SiO_2_ (300 nm) and PTFE (900 nm)	0.35	Experimental^2^	0.2	19.4	1.27	2.5
(W2)	33	1.0	SiO_2_ (400 nm) and Cytop (400 nm)	1.15	Experimental^4^	1.0	19.4	6.76	11.8
(W3)	33	1.0	SiO_2_ (400 nm) and Cytop (120 nm)	1.80	Experimental^4^	1.3	19.4	8.26	11.8

As shown in Table 1, the experimental efficiency may deviate from the numerical efficiency by up to 50%. Since we used experimental surface areas to predict the DEG efficiency, the deviation must originate from the electrical model (Eq. (20)). Even though this equivalent circuit model^2^ is remarkably accurate for low currents and stands on solid physical grounds, it was not validated for higher currents nor used to predict efficiencies in previous works. Like many physical models, it may fail to resolve singularities such as the current spike upon contact, or possibly nonlinear electro-hydrodynamic couplings or electrical double layer effects during fast discharges. We also note that these very fast current dynamics are difficult to capture experimentally which suggests that an accurate comparison would be very challenging. Nonetheless, the general trend between different DEG architectures is well conserved, which suggests that these calculations may be a useful guide for DEG design.

According to Fig. 5a, the conversion efficiency of impinging droplets depends on the droplet size and impact speed. The impact speed is set as initial condition in the simulations and controlled by the falling height in the experiments^18^. For large droplets impacting at high speed (X5), most of the energy (83%) is lost as viscous work, in good agreement with Fig. 3 and previous studies^15^. The recoil consumes less than 2% of the total energy ${{\mathcal{E}}}_{\max }$ by itself. The second largest loss contribution (≃10% of the total, 74% of the energy available after recoil) is the difference between the energy after recoil and the electrical energy. This large mismatch is shared across all sizes of droplets regardless of the impact speed, and represents the mechanical energy ${{\mathcal{E}}}_{\infty }$ remaining after the droplet detaches from the electrode. By analogy with bouncing droplets^21,29^, it is likely that this energy is a combination of internal kinetic energy and surface vibration energy. Next, an optimized load allows extracting almost 75% the electrical energy (resistive losses are negligible and the static losses represent ~1% of ${{\mathcal{E}}}_{\max }$).

Having identified that most of the energy is dissipated by viscous work and inefficient energy conversion during recoil, we now point to some ways to reduce these losses. The ratio of viscous to capillary work scales as ${\rm{Ca}}=\frac{\mu {U}_{0}}{\gamma }$ and looks insensitive to the main dimensions of the surface, which suggests that micropatterns which were successful at reducing the droplet spreading and contact time^38^ may not help minimizing the viscous dissipation. However, our simulations suggest that decreasing the impact velocity from 1.7 m/s (X5) to *U*_0_ = 0.1 m/s (X1) can cut the viscous dissipation during spreading to only 15% of ${{\mathcal{E}}}_{\max }$. This is in line with the improvement of restoring coefficient of bouncing Leidenfrost-levitated droplets at lower speed^21^. Therefore, while high-speed impacts generate more energy, low-speed impacts convert kinetic to surface energy with a much higher efficiency. Nonetheless, low-speed impacts then fail to extract the remaining mechanical energy from the droplet, so that only 5.1% of ${{\mathcal{V}}}_{\max }$ is converted to electricity when *U*_0_ = 0.1 m/s (X1) whereas up to 24% of ${{\mathcal{V}}}_{\max }$ becomes electricity when *U*_0_ = 1.7 m/s (X5).

A tentative interpretation is based on the following rough estimate of the energy conversion^2^:

27 $${{\mathcal{W}}}_{L}\simeq \frac{{({{\Delta }}A)}^{2}{\sigma }^{2}}{{A}_{\max }{c}_{p}}$$

with Δ*A* the difference of liquid-solid surface area between the time when the droplet connects to the electrode and when it breaks away from it. Note that this estimate differs from the ${E}_{0}={A}_{\max }{\sigma }^{2}/(2{c}_{p})$ proposed by Wu et al.^2^ by a factor ${\left(\frac{{{\Delta }}A}{{A}_{\max }}\right)}^{2}$, which allows discussing the irreversibility of the droplet motion. At high impact speed, the liquid motion is irreversible which results in a large Δ*A*, whereas at low impact speed, the flow motion is essentially reversible so that Δ*A* becomes very small which ruins the device overall efficiency.

Similarly, according to Eq. (27), reducing the surface capacitance or increasing the surface charge^4^ increases the ratio of electrical to capillary energy from 5.5% (W1) to 35% (W3) as shown in Fig. 5b. Therefore, the energy efficiency of DEG devices could be increased by reducing the impact speed to minimize viscous losses. This will result in a larger share of energy escaping during the droplet rebound (${{\mathcal{E}}}_{\infty }$) which may be minimized by increasing the substrate charge.

A related issue to DEG energy generation is the manufacturing of DEG devices and the pre-charge needed before they can operate at full efficiency. In this paper, we restricted the discussion to the steady-state regime because we believe that the technology is too immature to discuss this point. For example, Xu et al. generator requires 16,000 impinging droplets^1^ (about 2.4 J) and Wu et al. charged their substrates for 15 min using homogeneous electrowetting-assisted charge injection (h-EWCI)^2,4,13^ which requires 1.5 mJ of electrostatic energy in theory, but nearly 81 kJ in practice due to the 90 W consumption of the voltage amplifier used in their study. We are hopeful that suitable material choice and optimized industrial setup will dramatically reduce these pre-charge energies.

In this paper, we have used a combination of analytical equations, numerical model and experimental data to map out the energy losses during DEG generation. The energy conversion efficiency of these devices is mainly limited by viscous dissipation and poor capillary to electrical energy conversion. Experimental data and our numerical simulations show that a small fraction of the initial kinetic energy of impinging droplets is converted into surface energy at maximum spread. The remaining energy is lost as shear work and internal kinetic energy. Even though slower impacts provide less peak power, they dissipate much less energy. For applications where the total energy is critical (as opposed to the peak power), slower impact velocities may allow extracting more energy. This may be achieved by cascading generators with small gap height between them, or even harvesting the energy of crawling droplets^39^. Furthermore, increasing the device charge is indeed a key element in the path of improving DEG efficiency. By reducing the impact speed and increasing the surface charge, it makes little doubt that DEG efficiency can be improved beyond the current 10%.

## Materials and methods

### OpenFoam simulations

Our simulation is performed in OpenFOAM and based on the case of breaking dam in case base of OpenFOAM^40^. The simulation area is 30 × 30 × 40 mm^2^, discretized into 120 × 160 × 120 elements. The fluid properties are shown in Table 2. We used a laminar flow solver and VOF explicit interface tracking.

**Table 2:** Simulation parameters

Parameters	Value
Air kinematic viscosity	1.48 × 10^−5^ m^2^/s
Air density	1.0 kg/m^3^
Water kinematic viscosity	1.0 × 10^−6^ m^2^/s
Water density	1.0 × 10^3^ kg/m^3^
Surface tension	7.0 × 10^−2^ N/m
Contact angle	114°

A simulation script is available in Supplementary Information.

## Supplementary information

Latex-article&SUPPLEMENTAL INFORMATION
