# Supplementary material for: Hydrodynamic constraints on the energy efficiency of droplet electricity generators
Source: Microsyst Nanoeng. 2021 Jun 21;7:49. doi: 10.1038/s41378-021-00269-8 (PMC8433426; doi:10.1038/s41378-021-00269-8)
Supplement: Supplementary file 1 — Latex-article&SUPPLEMENTAL INFORMATION [file 41378_2021_269_MOESM1_ESM.pdf]

Supplementary information for:
"Hydrodynamic constraints on the energy efficiency of droplet electricity generators"

Antoine Riaud,* Cui Wang, and Jia Zhou
State Key Laboratory of ASIC and System, School of Microelectronics, Fudan University, Shanghai 200433, China

Wanghuai Xu and Zuankai Wang
Department of Mechanical Engineering, City University of Hong Kong, Hong Kong 999077, China†

(Dated: April 26, 2021)

I. ANALYTICAL SOLUTION OF WU'S MODEL

The charge q driven through the load by the droplet motion:

$$\frac{dq}{dt} = \frac{1}{Rc_p}\left(\sigma - \frac{q}{A}\right), \tag{S1}$$

with σ the surface charge of the polymer, c_p the capacitance of the polymer and $R = R_L + R_D$ the total resistance of the circuit, including the droplet resistance R_D and the load R_L. In Wu's model, $A(t)$ stands for the evolving area of the droplet, but the overlap area of charged polymer in contact with the droplet should be used instead when the polymer charge is non-uniform. Eq. (S1) is linear ordinary differential equation of the first order. A generic solution reads $q = Bq_H + q_P$ with q_H the solution of the homogeneous equation $Rc_pA\frac{dq_H}{dt} + q_H = 0$, q_P a particular solution of Eq. (S1) and B a constant determined from the initial conditions.

The solution q_H of the homogeneous equation reads:

$$q_H = \exp\left(-\int_0^t \frac{1}{Rc_pA(s)}ds\right). \tag{S2}$$

Using the variation of the constant, a particular solution q_P of Eq. (S1) reads:

$$q_P = q_H(t)\mathcal{C}(t) \tag{S3}$$

$$\text{with: } \mathcal{C} = \frac{\sigma}{Rc_p}\int_0^t \frac{1}{q_H(s)}ds. \tag{S4}$$

Since $q_H(0) = 1$ and $q_P(0) = 0$, the initial condition $q = 0$ at the droplet contact sets $B = 0$, so the charge reads $q = q_P$. From Eq. (S2), we note that $q_H \geq 0$ for all times t, therefore $\mathcal{C}$ is always of the same sign as σ and so is $q(t)$.

II. TIME STAMPS OF THE OPENFOAM SIMULATION RESULTS

Tab. S1: Simulation parameters.

Impact velocity (m/s)	0.1 m/s	0.5 m/s	0.7 m/s	1.0 m/s
Time (ms)	7	5	3	3
	15	15	13	9
	25	25	23	17
	37	37	35	23

III. COMPARISON OF IMPACT DYNAMICS OF 100 MM NACL WATER SOLUTION AND DEIONIZED WATER

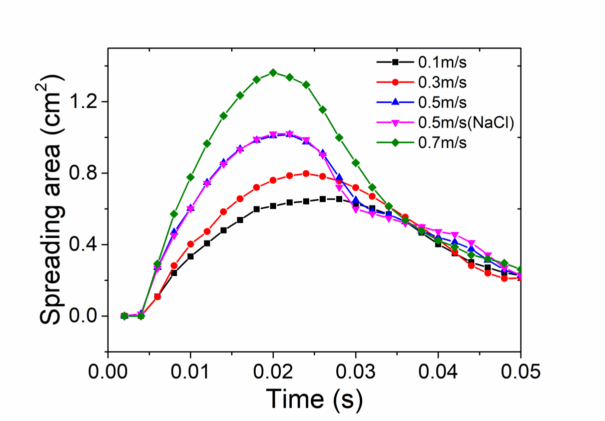

Fig. S1. Surface area of impacting droplets. Droplets of 100mM NaCl are shown together with DI water (0.5 m/s). The salt solution density is 1004 kg/m^3 and its viscosity is 1 mPa.s. Other parameters are the same as in table 2 of the manuscript.

* antoine_riaud@fudan.edu.cn; http://homepage.fudan.edu.cn/ariaud/ † zuanwang@cityu.edu.hk
